# Pan-cancer Analysis of NEDD4L and Its Tumor Suppressor Effects in Clear Cell Renal Cell Carcinoma

**DOI:** 10.7150/jca.58004

**Published:** 2021-08-28

**Authors:** Huiyue Dong, Ling Zhu, Jingjing Sun, Yi Zhang, Qiang Cui, Lin Wu, Shushang Chen, Jun Lu

**Affiliations:** 1 Fujian Provincial Key Laboratory of Transplant Biology, Fuzong Clinical College, Fujian Medical University, Fuzhou 350025, China; 2 Laboratory of Basic Medicine, Dongfang Hospital (900 Hospital of the Joint Logistics Team), Xiamen University, Fuzhou 350025, China; 3 Department of Urology, 900 Hospital of the Joint Logistics Team, Fuzhou 350025, Fujian, China; 4 Nephrology and Urology Department, The Second Affiliated Hospital of Wannan Medical College, Wuhu, China.

**Keywords:** NEDD4L, pan-cancer, TCGA, immune cell infiltration, ubiquitination

## Abstract

The expression level of NEDD4L, an E3 ubiquitin ligase, has changed significantly in human cancers. In this study, we aimed to study the expression of NEDD4L in pan-carcinoma and its function in malignant tumors. We analyzed the gene expression level of NEDD4L in pan-cancer from The Cancer Genome Atlas (TCGA) microarray data set, the correlation between gene expression and overall survival, disease-specific survival, and tumor immune microenvironment changes. NEDD4L expression changes in half of the cancer types. Low expression of NEDD4L gene predicts poor overall survival and disease-specific survival (DSS) in renal clear cell carcinoma (KIRC) and renal chromophobe cell carcinoma (KIRP). NEDD4L is negatively related to interstitial cell infiltration and immune cell infiltration in most common cancers. Furthermore, the low expression of NEDD4L was verified in our clear cell renal cell carcinoma (ccRCC) clinical tissues. In ccRCC cells, NEDD4L overexpression significantly reduced cell proliferation and migration. In the functional analysis, we proved that NEDD4L could inhibit ERBB3 and MAPK signaling pathways. When cells are deficient in nutrition, NEDD4L promoted the degradation of the autophagy regulatory protein ULK1. Our study provides novel insights into the role of NEDD4L in pan-cancer. NEDD4L may play a tumor suppressor effect in ccRCC, through tumor immune regulation and ubiquitination of key intracellular kinases.

## Introduction

Ubiquitination mediated by the ubiquitin-proteasome system is an important process of post-translational modification of biological proteins, and plays a very important role in protein localization, metabolism, regulation and degradation 1. Studies have found that the ubiquitin-proteasome system can regulate the biological functions of tumor cell proliferation and metastasis by mediating the degradation of a variety of tumor-related proteins 2. The key enzymes involved in this system include ubiquitin activating enzyme E1, ubiquitin conjugating enzyme E2 and ubiquitin ligase E3 3. Different ubiquitin ligase E3 mediates the transfer of activated ubiquitin from the conjugate enzyme E2 to the substrate, and acts on different substrate proteins, which determines the specificity of ubiquitination modification. The substrate specificity of ubiquitin ligase E3 makes it considered a good target for cancer treatment.

NEDD4L (Neural Precursor Cell Expressed Developmentally Down-Regulated 4-Like), also known as NEDD4-2, is a member of the neural precursor cell expression down-regulated factor 4 (NEDD4) protein family. The expression level of NEDD4L has also changed significantly in human cancers. In gallbladder cancer cells, the expression level of NEDD4L was significantly up-regulated compared with normal epithelial cells 4. At the same time, NEDD4L is generally highly expressed in melanoma cells 5. However, in prostate cancer specimens, the expression of NEDD4L was significantly reduced compared with neighboring normal tissues 6. It is reported that NEDD4L expression level is significantly down-regulated in non-small cell lung cancer 7. Down-regulation of NEDD4L expression enhances the aggressiveness of NSCLC tumors 8. At the same time, NEDD4L low expression group has a shorter survival time than non-low expression group. Similar observations have also been reported in colorectal cancer 9. NEDD4L is related to the regulation of many central pathways in cancer, including TGF-β, Wnt and EGFR signaling pathways 9, 10.

The Cancer Genome Atlas (TCGA) contains data on 33 cancers from more than 10,000 patients 11. The expression of NEDD4L in the data of TCGA pan-cancer species, its correlation with patient survival, the main function of genes, and whether it will affect the tumor immune microenvironment and other issues are in this paper through TCGA data analysis to draw conclusions. Select a tumor type whose NEDD4L gene expression significantly affects the prognosis of patients, and focus on how NEDD4L affects the malignant phenotype of tumor cells.

## Materials and Methods

### Data obtained from UCSC Xena Databases

We downloaded gene expression RNASeq (HTSeq-Fragments Per Kilobase per Million (FPKM)) data, somatic mutation data (varscan2_snv.tsv.gz), survival data and an integrated TCGA pan-cancer clinical resource (TCGA-CDR) from UCSC Xena Databases (http://xena.ucsc.edu/).

### Pan-Cancer NEDD4L Expression Profile Analysis

We used a Perl script to generate a txt file of NEDD4L gene expression. Gene expression summary was performed by a R package “ggpubr”. NEDD4L protein expression summary among cancer types was obtain from The Human Protein Atlas (www.proteinatlas.org).

### Survival Analysis of NEDD4L Expression

According to the median expression value of NEDD4L, we divided the patients in each cohort into "high" and "low" groups. The Kaplan-Meier method is used to estimate the survival function, and the R software package is used for overall survival analysis and the corresponding visualization "survival" and "survminer". Disease-related survival analysis and forest plots of all tumors are visualized using R packages "survival", "survminer", and "forestplot".

### Gene Set Enrichment Analysis (GSEA) analysis of KIRC

The c2.cp.kegg.v7.1.symbols.gmt and c5.all.v7.1.symbols.gmt files are downloaded from the molecular signature database (MSigDB, http://software.broadinstitute.org/gsea/index.jsp). These two files were used for GSEA analysis. According to the median expression of NEDD4L gene in renal clear cell carcinoma samples, we divided them into high expression group and low expression group. Gene Ontology (GO) and Kyoto Encyclopedia of Genes and Genomes (KEGG) pathway analysis were completed by using the clusterprofiler R package. According to the default weighted enrichment statistical method, this process is repeated 1,000 times for each analysis. According to p value < 0.05, false discover rate (FDR) < 0.25 and p value < 0.05, FDR < 0.05 were used to screen statistically significant pathways and biological processes.

### The relationship between NEDD4L gene expression and tumor microenvironment

The content of stromal cells and total immune cells in tumor tissues is realized by Pearson correlation analysis, which is realized by R package "estimate" analysis 12. Only the tumor sample data in TCGA-kidney renal clear cell carcinoma (KIRC) is retained. CIBERSORT's (cell type identification by estimating relative subsets of known RNA transcripts) algorithm has been used to evaluate the composition of immune cells in many cancer types 13. The content of different types of immune cells in KIRC tumor tissues is achieved by "CIBERSORT.R", p Value < 0.001. Relevant pictures are visualized by R package "ggplot2" "ggpubr" "ggExtra".

### Clinical specimen

From November 2013 to November 2015, 50 cases of renal clear cell carcinoma and matched adjacent tissues (> 2 cm from the edge of the cancerous tissue) were selected from the renal cancer patients in Fuzhou General Hospital. The patients were 28-77 years old, with an average age of 55.5 years. All cancer tissue specimens were confirmed to be clear cell renal cell carcinoma (ccRCC) by pathology. The isolation and use of human tissues were approved by the Human Research Ethics Review Committee of Fuzhou General Hospital (approval number 2013-017). All patients provided written informed consent. The pathological classification was verified according to Furhman classification. The details were described in our previous study 14, 15.

### Cell culture

ACHN and 786-O cells were provided by Shanghai Jikai Gene Technology Co., Ltd. (Shanghai, China). Cells are routinely checked for mycoplasma contamination every 2-3 months. ACHN and 786-O cells were cultured in RPMI-1640 medium (Thermo Fisher Scientific, Inc., Waltham, MA, USA) containing 10% (v/v) fetal bovine serum (Thermo Fisher Scientific, Inc.) and 1% penicillin/streptomycin (Thermo Fisher Scientific, Inc.). All the cells were cultured at 37 °C in a humidified incubator under 5% CO_2_.

### Quantitative polymerase chain reaction

Total RNA was extracted from tissues using Total RNA Kit II (Omega Bio-Tek, Inc., Norcross, GA, USA). After RNA quantification, 3 μg of total RNA was reverse transcribed into cDNA using the RevertAid First Strand cDNA Synthesis kit. PowerUp SYBR Green mix (Applied Biosystems, Thermo Fisher Scientific, Inc.) is used for quantitative polymerase chain reaction (qPCR). Use NEDD4L and β-actin primers to perform qPCR on cDNA: NEDD4L forward primer, 5'-CAGGGTCCAGAAGCAGATGA-3' and reverse primer, 5'-GAGTAGCACAGCCTTCCAGA-3'; α-actin forward primer 5'-TGACGTGGACATCCGCAAAG-3' and reverse primer 5'-CTGGAAGGTGGACAGCGAGG-3'. PCR was carried out for 40 cycles under the following conditions: 30 seconds at 94 °C and 60 seconds at 58 °C. By using the formula 2-∆∆Ct to normalize β-actin mRNA, the relative fold change of mRNA expression is calculated 16.

### Lentiviral and siRNA transfection

The lentivirus pLVX-Puro-NEDD4L and the control virus pLVX-Puro were purchased from a public protein/plasmid library (PPL, Nanjing, China). For virus infection, the virus is diluted with serum-containing cell culture medium containing 8 μg/mL polyethylene. Change to fresh medium after 12 hours. Then puromycin (1 μg/mL, Sigma) was added to select stable cells (10 days).

NEDD4L silencing was performed using one custom made siRNAs targeting NEDD4L mRNA region (si-NEDD4L sense: CCUCUGUAAUGAGGAUCAUUUTT, si-NEDD4L antisense: AAAUGAUCCUCAUUACAGAGGTT) and one scramble control siRNA (HippoBio, China) and transfected by Lipofectamine RNAiMAX (Thermo Fisher Scientific, Inc.). Transfection Reagent followed the manufacturer's instructions.

### Water-soluble tetrazole (WST)-1 detects cell proliferation

Take the cells of the experimental group and the control group, adjust the cell number to 2×10^4^ cells/ml, and inoculate 100 μL of cells into a 96-well cell culture plate. After 24, 48, and 72 hours of adherence to the cells, 10 μL of water-soluble tetrazole (WST-1) reagent (Roche, Mannheim, Germany) was added, and incubated for 45 minutes. Then, a spectrophotometer (Multiskan GO, Thermo Scientific) was used to measure the optical density (D450) of each hole at a wavelength of 450 nm. Four repeat holes are set for each group.

### NEDD4L integrated network analysis

In order to gain more insights into NEDD4L functionality. We used UbiBrowser (http://ubibrowser.ncpsb.org/ubibrowser/home/index) to predict the ubiquitination substrate of E3 ubiquitinase, NEDD4L, and obtained data from BioGRID (https://thebiogrid.org/) for molecules that interact with NEDD4L. Pathway Commons (https://www.pathwaycommons.org) is used for multi-gene interaction analysis. STRING (https://string-db.org/) is used for multi-gene function prediction.

### Immunohistochemical staining

A tissue microarray (HKidCRC060CS01; Shanghai Outdo Biotech Co., China) was constructed with formalin-fixed paraffin-embedded 22 paired ccRCC and adjacent non-tumor renal tissues. Five of these pairs have distal normal renal tissues. The paraffin-embedded sections were stained with antibodies against NEDD4L (dilution at 1:500; HPA024618) at 4 °C overnight and incubated with secondary antibody. Negative controls were stained with isotype control IgG. Then, the sections were counterstained with hematoxylin and eosin. Immunohistochemical staining intensity was based on the proportion of cell staining and scored from 0 to 3 following criteria described previously 17. The slides were analyzed by standard light microscopy.

### Western Blot

For each group of proteins quantified by bicinchoninic acid (BCA), sodium dodecyl sulfate polyacrylamide gel electrophoresis (SDS-PAGE) was performed at 50 μg/well. After electrophoresis, the cells were transferred to the polyvinylidene fluoride (PVDF) membrane under a constant pressure of 60 V for 180 minutes. The blocking solution was blocked for 1 hour and reacted with the primary antibody overnight at 4 °C. Tris-Buffered Saline Tween-20 (TBST) was washed three times; horseradish peroxidase (HRP)-labeled goat anti-rabbit antibody was reacted at room temperature for 1 h, and TBST was washed 4 times; electrogenerated chemiluminescence (ECL) was added, exposed on X-ray film, film development, fixing, washing with water, drying, storage and photographing. NEDD4L, MEK, ERK, phospho-MEK, phospho-ERK, ERBB3, p62, LC3, ULK1, ACTB (β-actin) antibodies were purchased from Cell Signaling Technology (Denver, Massachusetts, USA). Horseradish peroxidase (HRP) labeled secondary antibody and ECL kit were purchased from Pierce (Thermo Fisher Scientific, Inc.).

### Statistical processing

Use SPSS13.0 software for statistical analysis. The data obtained are expressed as mean ± SD. The single-factor repeated measures analysis of variance found a statistically significant difference when p value < 0.05.

## Results

### Pan-cancer expression landscape of NEDD4L

**Figure 1:**
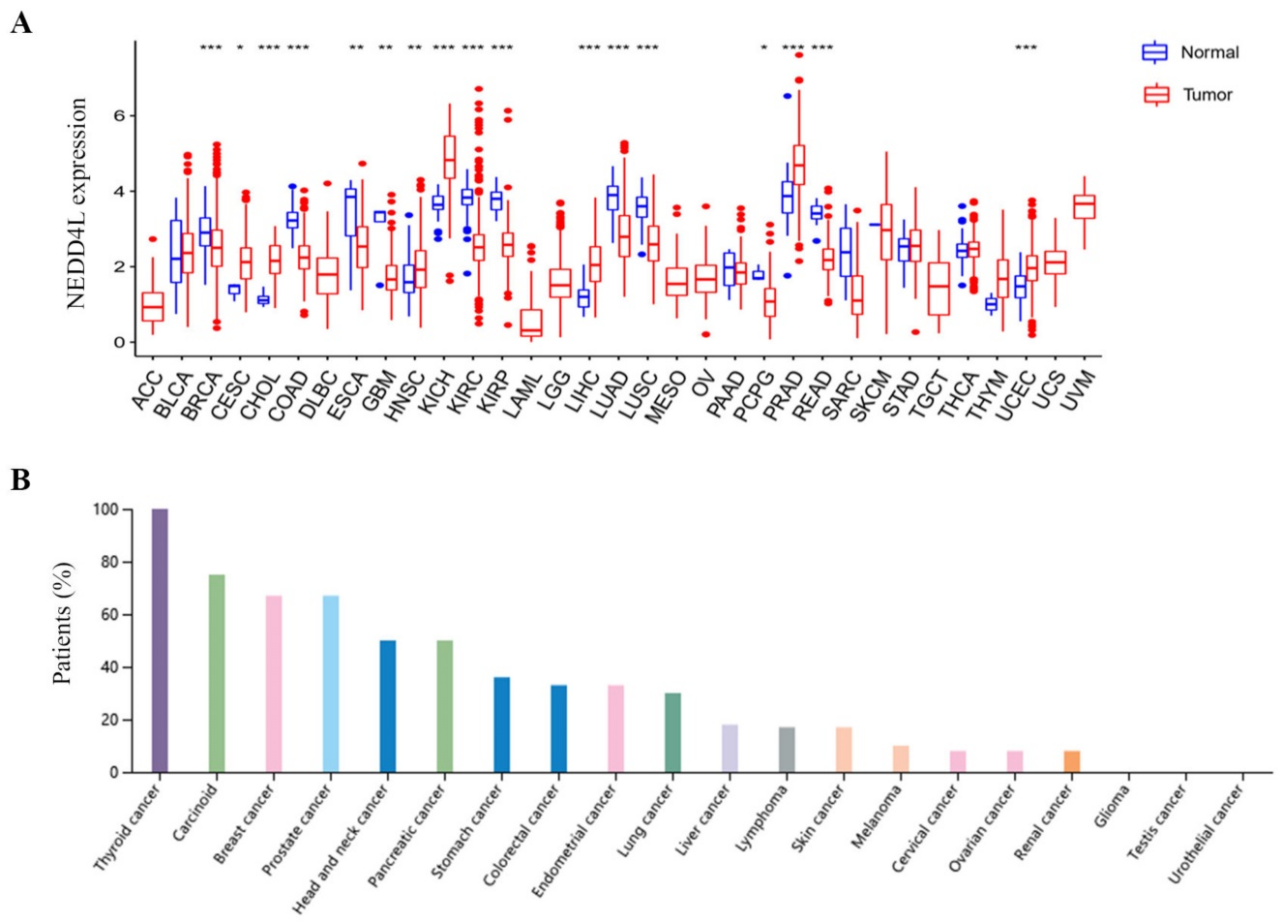
Analysis of NEDD4L expression. (A) Box plots of NEDD4L mRNA levels in neoplastic tissues. Blue represents non-tumor tissues and red represents tumor tissues. * p-value < 0.05, ** p-value < 0.01, ***p-value < 0.001. (B) Bar charts of NEDD4L expression analysis (immunohistochemical staining) in various cancers. The results were download from The Human Protein Atlas.

Among all 33 cancer types in the TCGA database, NEDD4L expression was significantly changed in 17 tumors, accounting for 51.52% of all cancer types. NEDD4L expression in cancer was significantly increased (according to cancer samples and normal samples) in cholangiocarcinoma (CHOL), kidney chromophobe (KICH), liver hepatocellular carcinoma (LIHC), prostate adenocarcinoma (PRAD) and uterine corpus endometrial carcinoma (UCEC), whereas, NEDD4L expression significantly reduced in breast invasive carcinoma (BRCA), colon adenocarcinoma (COAD), KIRC, kidney renal papillary cell carcinoma (KIRP), lung adenocarcinoma (LUAD), lung squamous cell carcinoma (LUSC) and rectum adenocarcinoma (READ) (Figure 1A, ***, p-value < 0.001). Figure 1B shows the protein expression rates of NEDD4L in patients of various cancers. Weak to moderate cytoplasmic positivity was observed in most cancers, such as thyroid, carcinoid, breast, and prostate cancers. Urothelial, testis, glioma and renal cancers were almost negative.

### NEDD4L expression and prognostic association

**Figure 2:**
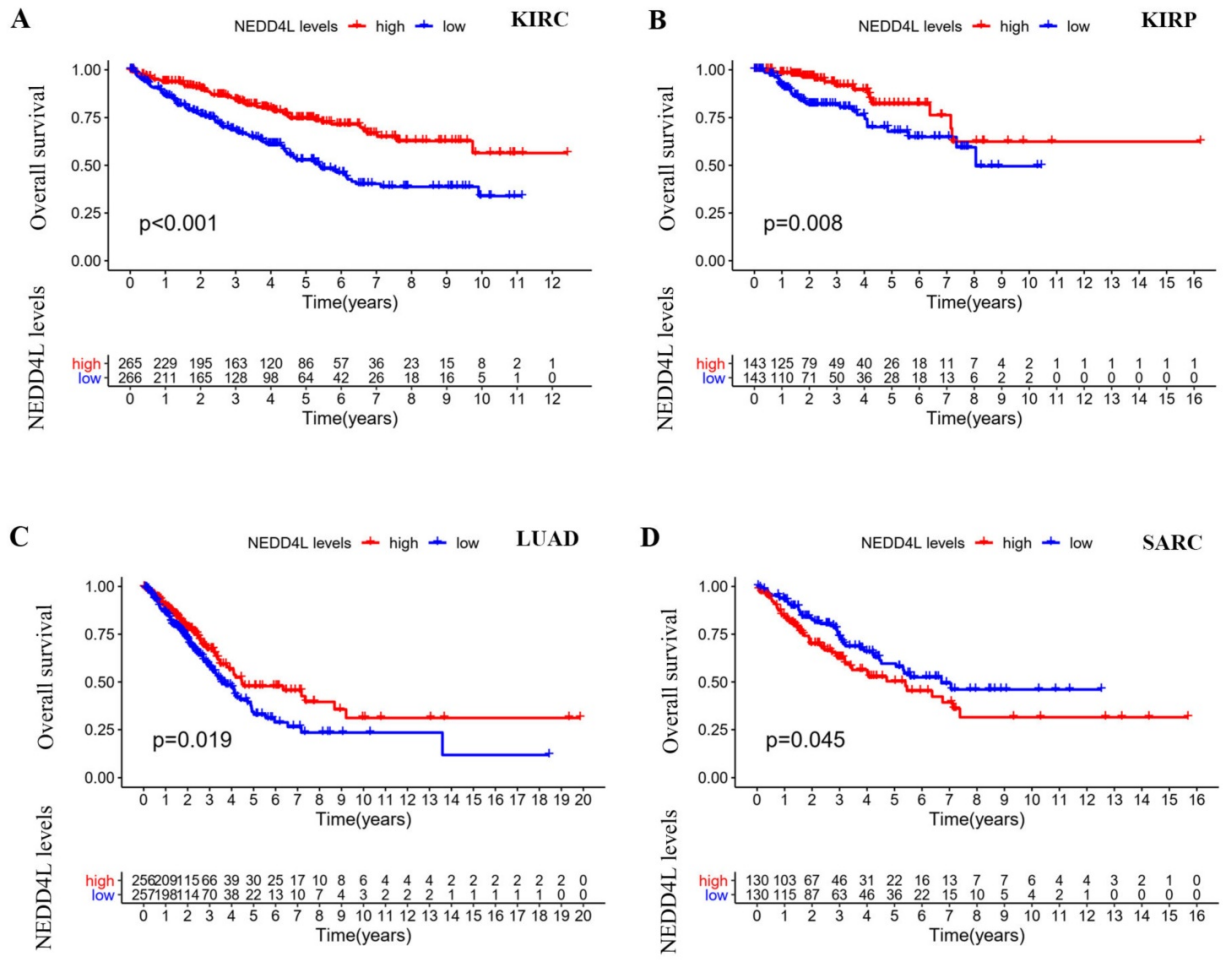
Overall survival analysis in TCGA cancers. (A-D) Kaplan-Meier curves represent NEDD4L association with patient overall survival in KIRC, KIRP, LUAD and SARC. p-value < 0.05.

**Figure 3:**
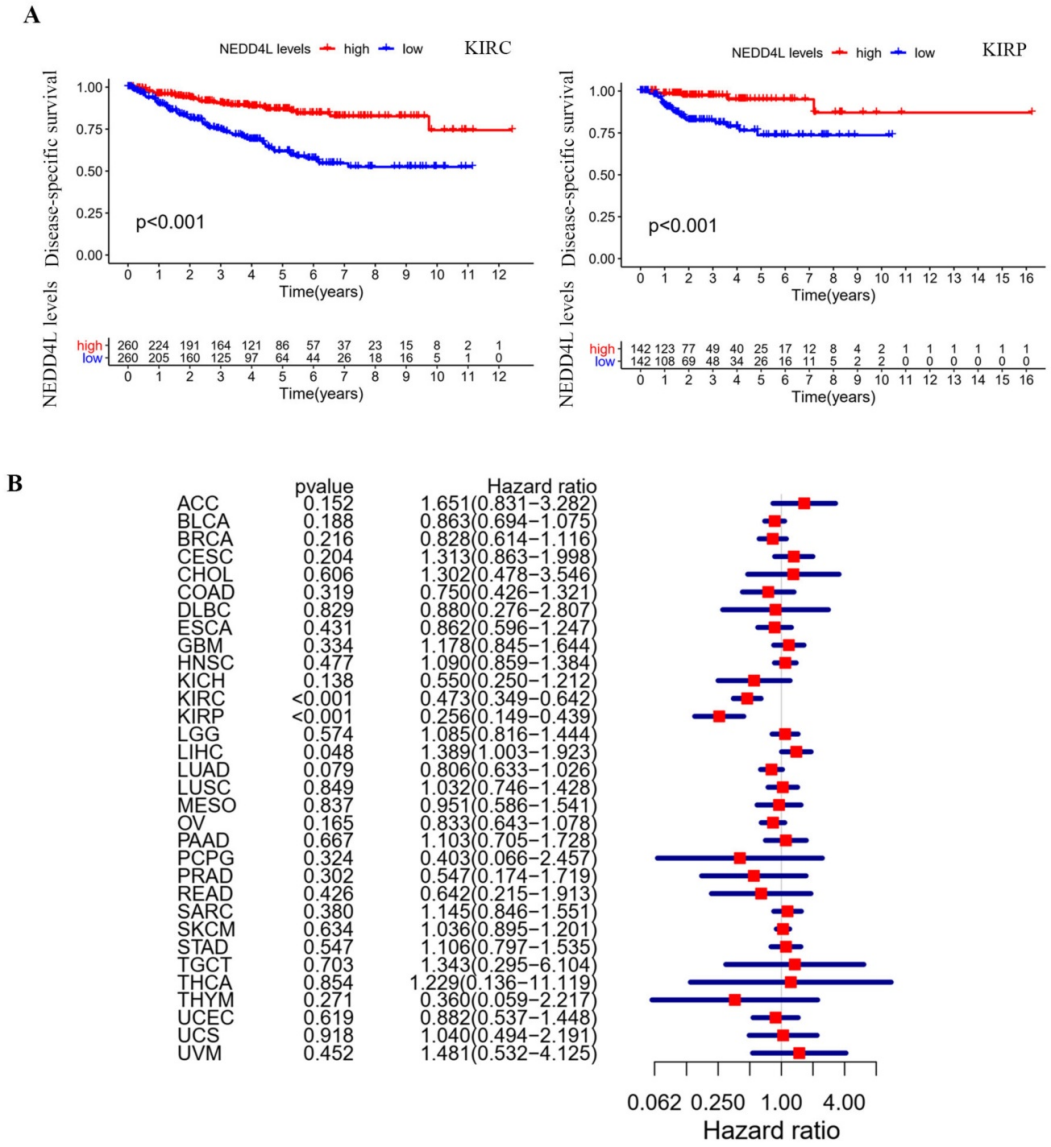
Disease-specific survival (DSS) analysis in TCGA cancers. (A) Kaplan-Meier curves represent NEDD4L association with DSS in KIRC and KIRP. p-value < 0.05. (B) Forest plot of the association of NEDD4L gene with DSS in each of cancers.

Kaplan-Meier plots 18 were used to evaluate overall survival (OS) and disease-specific survival (DSS). The DSS forest diagram is used to illustrate the correlation between NEDD4L gene expression and DSS in various cancer types in the TCGA database. Among the 33 cancer types in the TCGA database, there were four cancer types whose expression of NEDD4L protein can affect the overall survival of patients: KIRC, KIRP, LUAD, and sarcoma (SARC). NEDD4L was low in KIRC, KIRP, and LUAD, and lower gene expression predicts poor patient survival (Figure 2A). Low expression of NEDD4L gene in KIRC and KIRP was correlated with worse DSS (KIRC: hazard ratio: 0.473, 95% confidence interval: 0.349-0.642; p-value< 0.001; KIRP: hazard ratio: 0.256, 95% confidence interval: 0.149 -0.439; p-value< 0.001) (Figure 3B). These results indicated that NEDD4L gene expression may be one of the prognostic factors of KIRC and KIRP.

### Pan-cancer analysis of NEDD4L expression and tumor microenvironment

**Figure 4:**
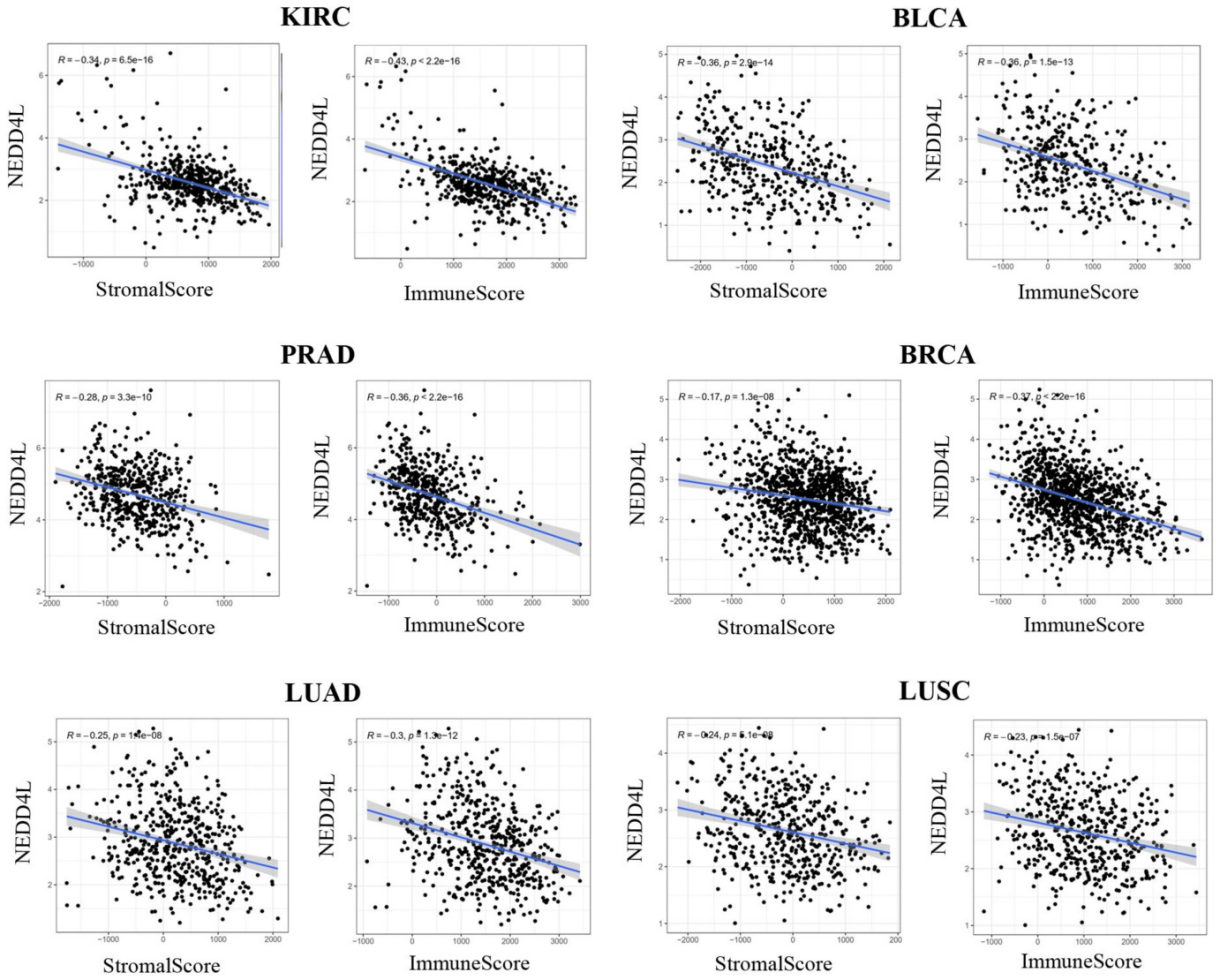
Correlations between expression level of NEDD4L and stromal cell or immune cell infiltration levels in KIRC, BLCA, PRAD, BRCA, LUAD and LUSC.

The tumor immune microenvironment plays a vital role in the development of many cancers 19, 20. ESTIMATE algorithm can infer the StromalScore (stromal cell infiltration level) and ImmuneScore (immune cell infiltration level) in tumor tissue. The correlation between NEDD4L gene expression and stromal cell infiltration levels or immune cell infiltration level in various types of tumor tissues has not yet been reported. In most cancer types (20 in 34 types, p-value <0.05), the expression of NEDD4L is negatively correlated with the infiltration value of interstitial cells and immune cells in tumor tissues (Table S1). Figure 4 shows that the top six cancer types with negative correlation are: KIRC, Bladder urothelial carcinoma (BLCA), PRAD, BRCA, LUAD and LUSC.

### The relationship between NEDD4L expression and immune cell infiltration in the TCGA-KIRC dataset

**Figure 5:**
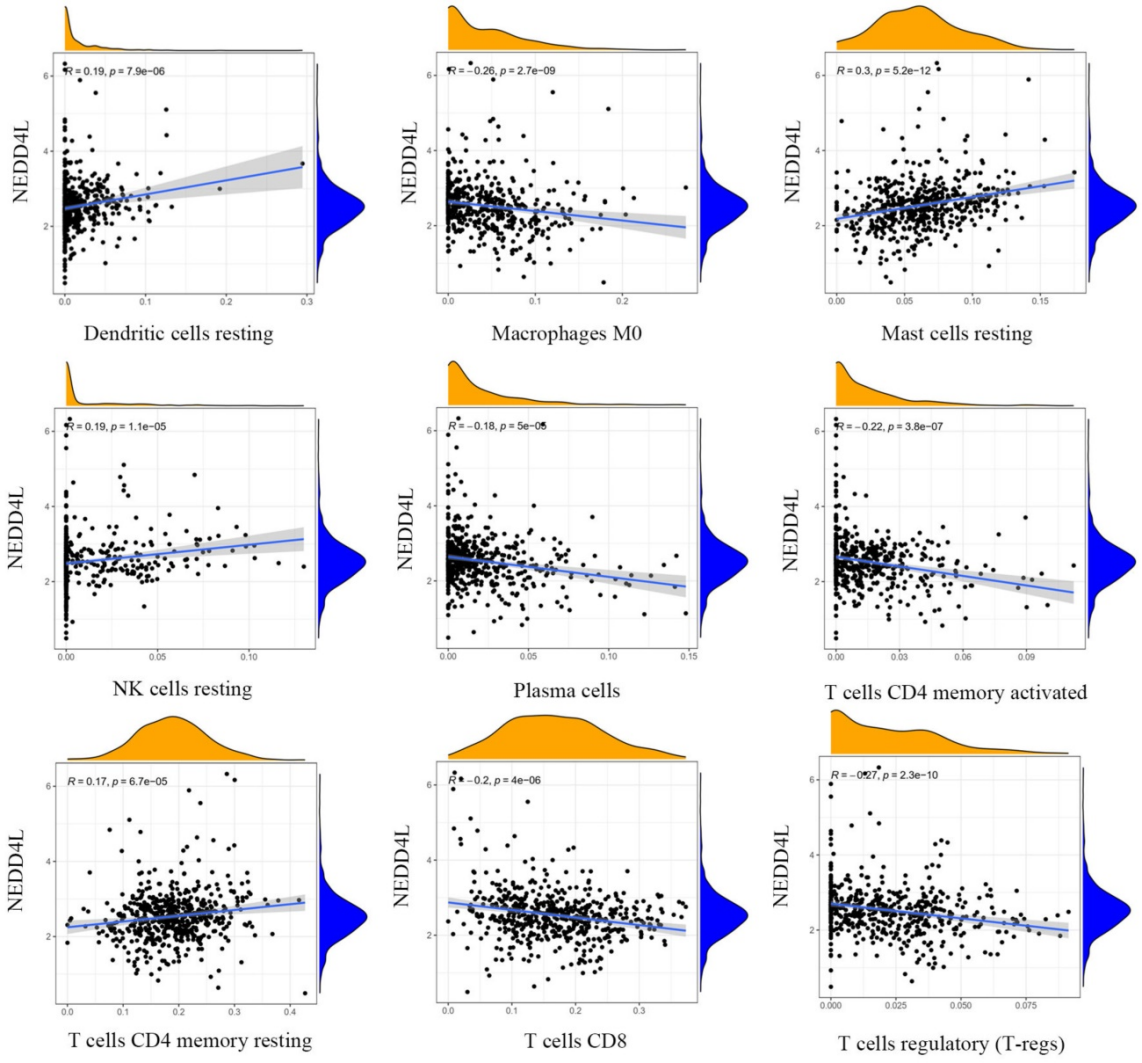
Correlations between expression level of NEDD4L and the infiltration levels of dendritic cells resting, macrophage M0, mast cells resting, NK cells resting, plasma cells, T cells CD4 memory activated, T cells CD4 memory resting, T cells CD8, and T-regs.

The results of gene expression and tumor microenvironment analysis indicate that NEDD4L may be closely related to immune function. There are a large number of lymphocyte infiltrations in tumor tissues, to further explore the influence of NEDD4L on the infiltrated patterns of specific lymphocyte types, using CIBERSORT, we investigated the relationship between the expression level of NEDD4L and the infiltration level of 22 immune cells in ccRCC (Figure 5). We found that NEDD4L expression is significantly negatively correlated with the ratio of three T cells, including cluster of differentiation 4 (CD4) memory activated T cells, CD8 positive T cells, regulatory T cells (T-regs), and positively correlated with CD4 positive T cells that have tumor-killing effects. T-regs are a type of CD4 + T cells that have powerful inhibitory functions. T-regs have been found to have an increased infiltration rate in many types of cancers, and a higher T-reg tumor infiltration rate indicates a poor clinical prognosis. In addition, most T cell are dysfunction in tumor tissues, such as only 10% of CD8-positive T cells have tumor recognition ability. These results suggest that NEDD4L may inhibit tumorigenesis and development by inhibiting the formation of tumor immune microenvironment.

### Gene function analysis of NEDD4L in KIRC dataset

As we shown NEDD4L is significantly lower in KIRC, and the low expression of NEDD4L can be used as a predictor of poor prognosis in KIRC. The function of NEDD4L in the occurrence of ccRCC is not clear, so we chose to study the role of NEDD4L abnormal expression in tumorigenesis in KIRC. TCGA-KIRC data is used to analyze NEDD4L gene set enrichment analysis (GSEA). The analysis results found that acetone metabolism, valine, leucine and isoleucine degradation pathways, and citrate cycle (TCA cycle) are positively correlated with NEDD4L expression levels. These metabolic pathways are down-regulated in ccRCC. Cytokine-cytokine-receptor interaction, leukocyte-activation, apoptosis pathway, etc. are negatively related to NEDD4L. These results indicate that NEDD4L may participate in tumor immune regulation and affect tumor cell growth and apoptosis.

### Overexpression of NEDD4L inhibits the growth and migration of renal clear cell carcinoma cell lines

**Figure 7:**
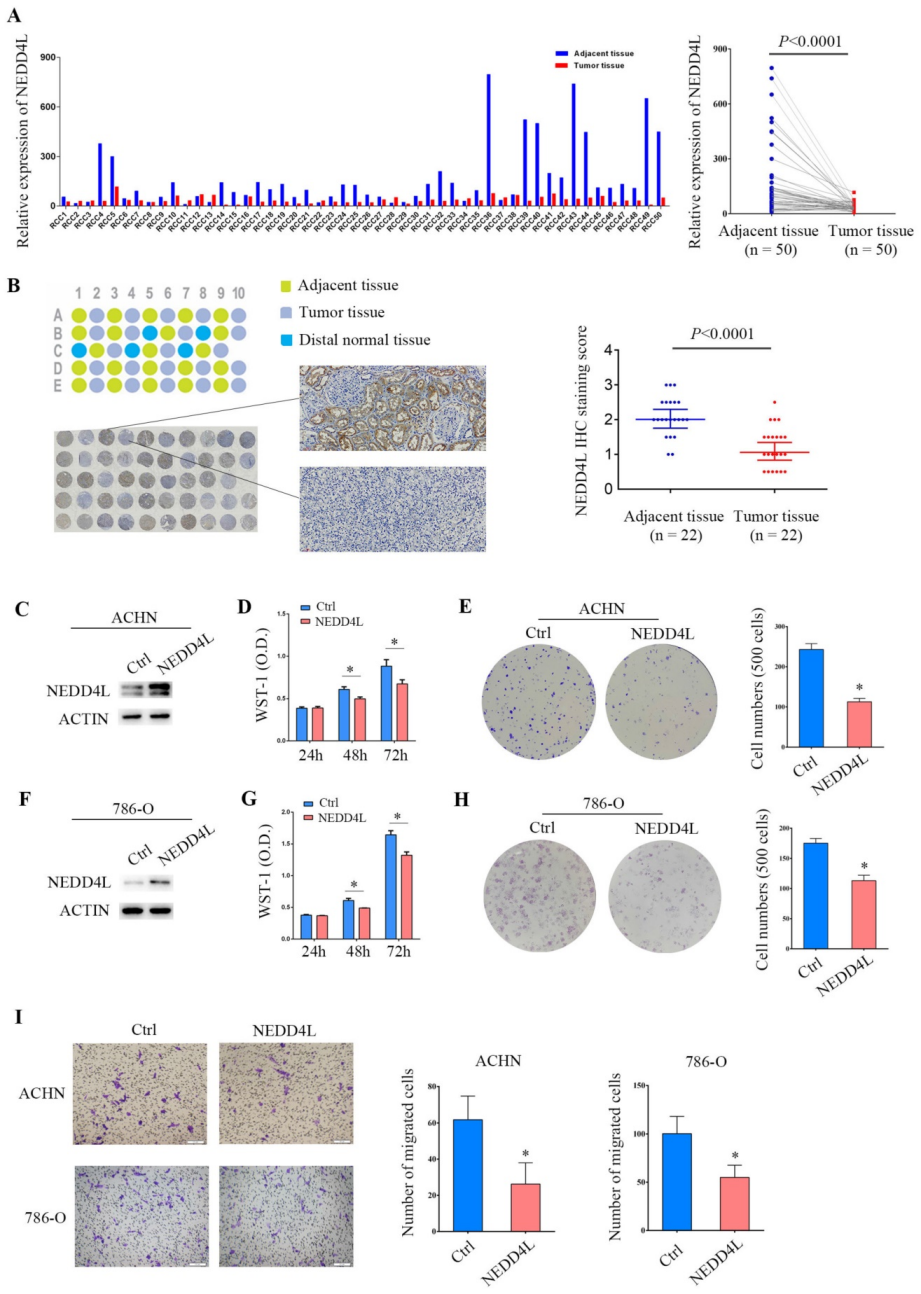
Effects of overexpression of NEDD4L on ccRCC cell growth. (A) 50 pairs of ccRCC samples were used to detect NEDD4L mRNA levels by quantitative-PCR. (B) Immunohistochemical detection of NEDD4L. There are 22 pairs of tissue samples in the tissue chip, 5 of which contain distal normal tissue controls. (C) ACHN cells stably transfected with NEDD4L lentivirus and performed by Western blot analysis. (D) The growth inhibition rates were measured by WST-1 assay kit at 24, 48, and 72 hours in ACHN cells that stably expressed NEDD4L. (E) Representative images of clonogenic assays of ACHN cells that stably expressed NEDD4L. (F) Western blot assay was performed in NEDD4L overexpressed 786-O cells. (G) WST-1 assay in 786-O cells that stably expressed NEDD4L. (H) Colony formation assay in 786-O cells that stably expressed NEDD4L. (I) Representative images of migration assays of ACHN and 786-O cells stably expressing NEDD4L and quantification of the relative migration cell number (right). Scale bar, 100 µm. All experiments were repeated three times. The *p*-value was measured using Student's *t*-test; * p < 0.05, compared with the control cells. Ctrl: cells transfected with empty control lentivirus.

From 2013 to 2015, our center collected 50 pairs of ccRCC tumor tissues and their adjacent tissues that were pathologically identified and surgically removed 14, 15. Quantitative PCR analysis showed that NEDD4L expression decreased in 82% of tumor tissues. Paired analysis showed that the level of NEDD4L mRNA in renal clear cell carcinoma tissue was significantly reduced (p <0.0001) (Figure 7A). A ccRCC paraffin tissue microarray was used for immunohistochemical detection. Results showed the protein levels of NEDD4L is also decreased in ccRCC tissues (p <0.0001) (Figure 7B).

Next, we tried to check the function of NEDD4L in ccRCC cells using a lentivirus-mediated overexpression system. It was confirmed that NEDD4L was highly expressed in ACHN cells (Figure 7C). The results of WST-1 determination showed that the proliferation of NEDD4L overexpression group was significantly lower than that of the control group at 48 and 72 hours after the cells grew adherently (p<0.05, Figure 7D). Compared with the control group, the ability of ACHN cells with stable NEDD4L expression to form colonies was significantly reduced (Figure 7E). These data were verified in another ccRCC cell line, 786-O cell line (Figure 7F-7H). In addition, in cells overexpressing NEDD4L, cell migration was significantly reduced (Figure 7I). These data collectively indicate that NEDD4L is a tumor suppressor of ccRCC.

### NEDD4L deregulates the stability of several protein kinases via ubiquitination activity in ccRCC

**Figure 8:**
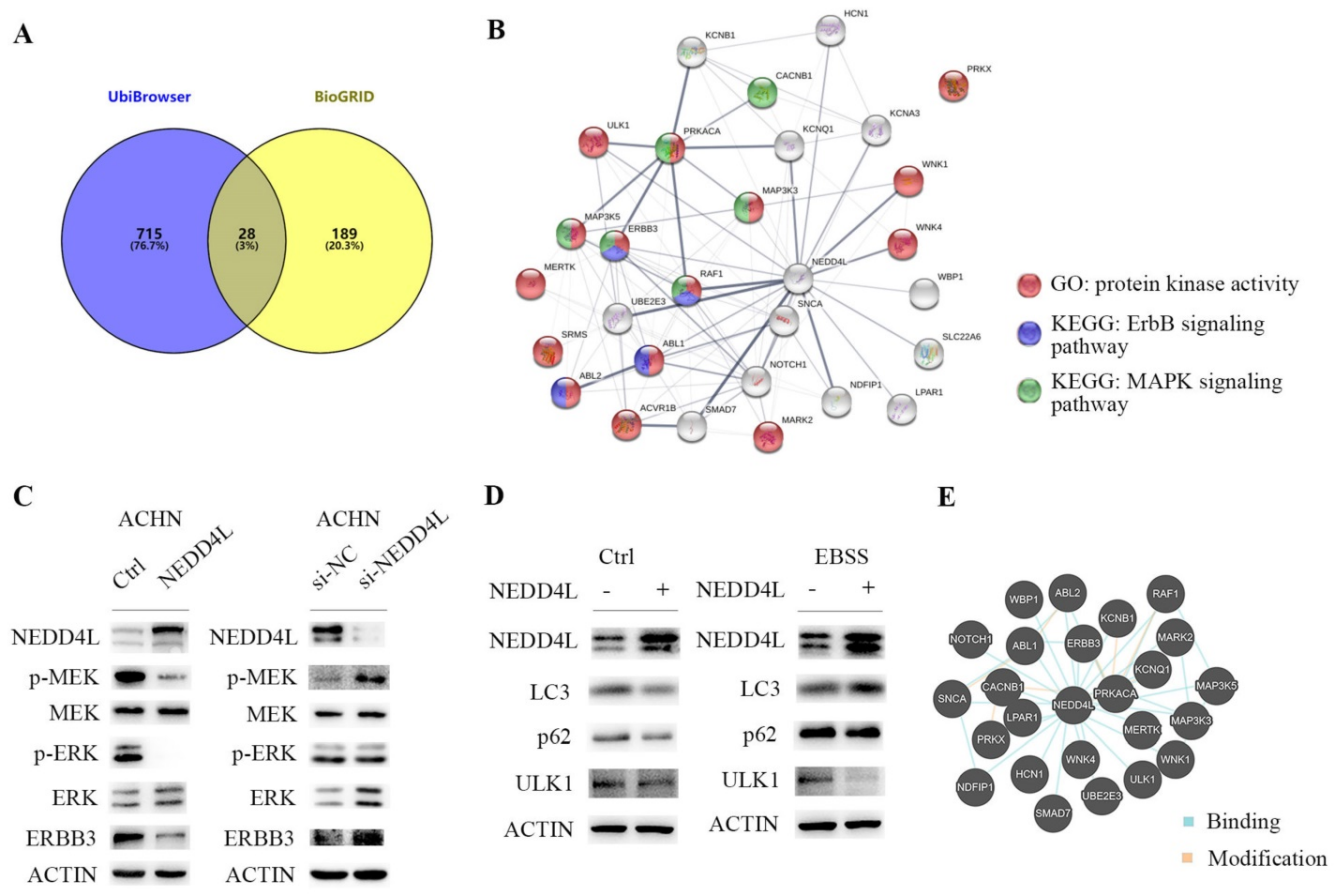
NEDD4L overexpression degrades the protein kinases. (A) Overlap of genes showing those both with predicted the ubiquitination substrates and confirmed interacting molecules of NEDD4L. (B) Biological network enrichment of 28 intersection genes predicted by STRING analysis. (C) Representative western blot analysis of NEDD4L overexpressed (NEDD4L), down-regulated (si-NEDD4L) or control cells. The levels of NEDD4L, phospho (p)-MEK, MEK, p-ERK and ERK, ERBB3 and β-actin (ACTIN) in ACHN cells were measured. The result is representative of three separate experiments. (D) NEDD4L overexpressed or control cells were plated overnight and subsequently treated with EBSS for 6 h and resolved in an SDS-PAGE gel. Samples were immunoblotted against NEDD4L, LC3, p62, ULK1 and ACTIN. (E) Protein-protein interactions network of 28 intersection genes predicted by Pathway Common.

NEDD4L is an E3 ubiquitinase 21 and there are many proteins regulated by it 22, 23. We used UbiBrowser to predict the ubiquitination substrate of E3 ubiquitinase, NEDD4L, and 743 molecules with the confidence score greater than 0.8. The data of molecules interacting with NEDD4L are obtained from BioGRID. There are 207 interacting molecules with evidence of ≥ 2 in BioGRID. Figure 8A shows 28 ubiquitinated substrates that interact with NEDD4L. STRING analysis results show that half of these genes have protein kinase activity (Figure 8B). Several genes are members of the ERBB3 or MAPK signaling pathway. In ACHN cells, NEDD4L is stably and highly expressed, and the phosphorylation levels of MEK and ERK in the cell are significantly reduced (Figure 8C), indicating that NEDD4L may degrade MEK upstream kinases through ubiquitination to inhibit the MAPK signaling pathway. ERBB3 is the direct ubiquitination substrate of NEDD4L 10, 24. In ACHN cells, high expression of NEDD4L could result in a decrease in MAPK pathway and ERBB3 protein, whereas down-regulated NEDD4L caused increased levels of MAPK pathway and ERBB3 protein (Figure 8C).

Protein kinase ULK1 has been reported to regulate cell autophagy and energy metabolism. When cell nutrition is deficient, ULK1 ubiquitination and degradation participate in autophagy regulation. NEDD4L is an E3 ubiquitinase involved in the regulation of ULK1 degradation during autophagy 25, 26. If NEDD4L is knockdown, the autophagy activity will be increased. In our ccRCC cell line, cells cultured by Earle's Balanced Salt Solution (EBSS) are deficient in nutrients and autophagy occurs. In cells with high NEDD4L expression, ULK1 is more easily degraded (Figure 8D). The results of protein interaction analysis in Pathway Common also showed that MAPK upstream kinases (MAP3K3, MAP3K5), ERBB3 and ULK1, which are important for tumor cell growth and proliferation, can bind to NEDD4L (Figure 8E). Our data indicate that in renal cancer cells, NEDD4L may inhibit tumor cell growth by ubiquitinating multiple kinase molecules.

## Discussion

In this study, we first analyzed the overall expression of NEDD4L in 33 cancer types in TCGA data and found that NEDD4L expression was abnormally changed in a variety of tumors. Subsequent overall survival analysis and disease-specific survival analysis found that low NEDD4L expression in KIRC and KIRP patients was associated with poor prognosis. Tumor immune microenvironment analysis found that NEDD4L gene expression is negatively correlated with stromal cell infiltration level and immune cell infiltration level in most cancer types. KIRC negative correlation is the most significant. Therefore, we focus on KIRC.

In the analysis of various immune cell ratios in the TCGA-KIRC data set, it was found that the low expression of NEDD4L may indicate an increase in the ratio of immunosuppressive T cells. GSEA gene function enrichment analysis results suggest that NEDD4L participates in tumor immune regulation in KIRC, and affects tumor cell growth and apoptosis. In our samples, we verified that NEDD4L expression decreased in ccRCC tissues. Two ccRCC cell lines ACHN and 786-O were infected with NEDD4L gene expression lentivirus. In cells stably overexpressing NEDD4L, cell proliferation slows down and the ability to form cell clones decreases. Transwell experiments found that the cell migration ability of NEDD4L overexpressing cells was inhibited.

NEDD4L is E3 ubiquitinase, which can regulate multiple signaling pathways in cells through the ubiquitination degradation pathway 22, 23. In previous studies, it has been shown that both NEDD4 and NEDD4L can bind to ERBB3 (HER3) to promote ubiquitination and degradation of ERBB3 10, 24. ERBB3 is an EGFR family protein, which is closely related to the occurrence and development and poor prognosis of breast cancer, ovarian cancer, prostate cancer, lung cancer and other tumors 27, 28. Uncoordinated 51-like kinase 1 (ULK1) plays a vital role in autophagy, initiating autophagy when starved 29, 30. ULK1 can also phosphorylate key glycolytic enzymes and maintain redox homeostasis 31. In the progress of autophagy, NEDD4L is involved in ULK1 ubiquitination and degradation 24. Lee et al. show that NEDD4L binds to ULK1, ULK1 expression was stable in NEDD4L knockdown in pancreatic cancer cells 25. In recent years, ULK1 disorders have been found in several human cancers 32. Our previous research results show that ULK1 is highly expressed in ccRCC, and ULK1 inhibitors can effectively inhibit the proliferation of ccRCC cells 33. Here, our results indicate that NEDD4L can also degrade ERBB3 in kidney cancer cells. NEDD4L can promote the degradation of ULK1 when cells in starvation condition.

Among the predicted NEDD4L ubiquitination substrates, there are several genes in the MAPK signal pathway, especially MAP3K3 are important kinases upstream of MAPK. In our study, it was found that the MAPK signal pathway was reduced in NEDD4L overexpressed renal carcinoma cells but activated in NEDD4L knockdown cells. This result preliminarily indicates that NEDD4L may inhibit the activation of MAPK pathway by ubiquitinating MAPK upstream kinase. MAP3K3 has been shown to participate in the growth and proliferation of a variety of tumor cells 34, 35. In future research, we will thoroughly explore the ubiquitination regulation effect of NEDD4L on MAP3K3.

Taken together, NEDD4L expression is abnormal in most common cancers. The low expression of NEDD4L predicts poor overall survival and disease-specific survival of KIRC and KIRP. NEDD4L may be involved in regulating the tumor immune microenvironment, and ubiquitinating and degrading key cell growth kinases to suppress the growth of renal cancer.

## Supplementary Material

Supplementary table S1.

## Figures and Tables

**Figure 6:**
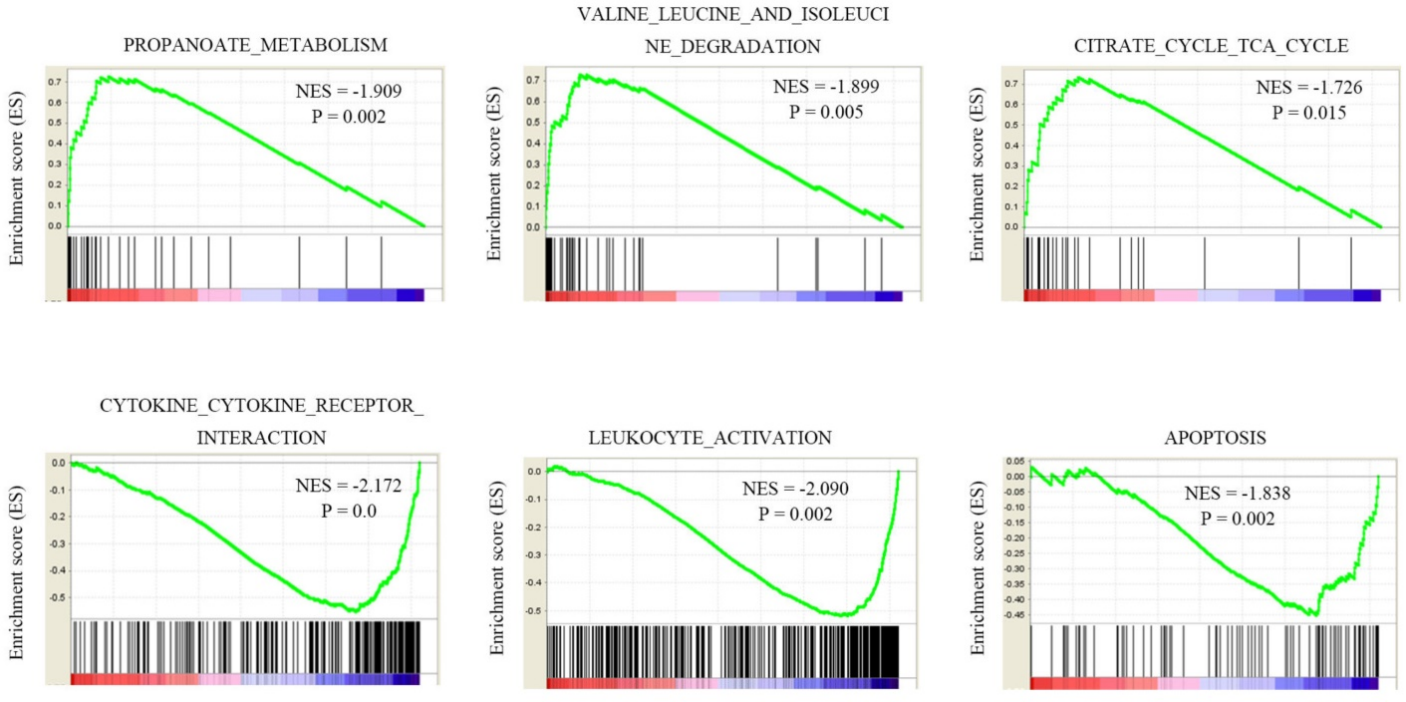
GSEA analysis of NEDD4L co-expression genes in TCGA-KIRC samples.

## Abbreviations

BCA: bicinchoninic acid
ccRCC: clear cell renal cell carcinoma
CD4: cluster of differentiation 4
GSEA: gene set enrichment analysis
GO: gene ontology
HRP: horseradish peroxidase
KEGG: kyoto encyclopedia of genes and genomes
PVDF: polyvinylidene fluoride
SDS-PAGE: sodium dodecyl sulfate polyacrylamide gel electrophoresis
TCGA: the cancer genome atlas
WST: water-soluble tetrazolium salt
ACC: adrenocortical carcinoma
BLCA: bladder urothelial carcinoma
BRCA: breast invasive carcinoma
CESC: cervical squamous cell carcinoma and endocervical adenocarcinoma
CHOL: cholangiocarcinoma
COAD: colon adenocarcinoma
DLBC: diffuse large B-cell lymphoma
ESCA: esophageal carcinoma
GBM: glioblastoma multiforme
HNSC: head and neck squamous cell carcinoma
KICH: kidney chromophobe
KIRC: kidney renal clear cell carcinoma
KIRP: kidney renal papillary cell carcinoma
LAML: acute myeloid leukemia
LGG: lower grade glioma
LIHC: liver hepatocellular carcinoma
LUAD: lung adenocarcinoma
LUSC: lung squamous cell carcinoma
MESO: mesothelioma
OV: ovarian serous cystadenocarcinoma
PADD: pancreatic adenocarcinoma
PCPG: pheochromocytoma and paraganglioma
PRAD: prostate adenocarcinoma
READ: rectum adenocarcinoma
SARC: sarcoma
SKCM: skin cutaneous melanoma
STAD: stomach adenocarcinoma
TGCT: testicular germ cell tumors
THCA: thyroid carcinoma
THYM: thymoma
UCEC: uterine corpus endometrial carcinoma
UCS: uterine carcinosarcoma
UVM: uveal melanoma
