# Supplementary material for: Pan-cancer Analysis of NEDD4L and Its Tumor Suppressor Effects in Clear Cell Renal Cell Carcinoma
Source: J Cancer. 2021 Aug 28;12(20):6242–53. doi: 10.7150/jca.58004 (PMC8425189; doi:10.7150/jca.58004)
Supplement: Supplementary file 1 — Supplementary table S1. [file jcav12p6242s1.pdf]

Table S1. Details of the infiltration value of interstitial cells and immune cells in tumor tissues

CancerType	Gene	StromalScore（p-value）	ImmuneScore (p-value)
KIRC	NEDD4L	6.48E-16	0
BLCA	NEDD4L	2.95E-14	1.50E-13
PRAD	NEDD4L	3.27E-10	3.32E-17
BRCA	NEDD4L	1.28E-08	0
LUAD	NEDD4L	1.37E-08	1.32E-12
LUSC	NEDD4L	5.11E-08	1.47E-07
UCEC	NEDD4L	1.69E-06	3.40E-07
SARC	NEDD4L	7.79E-07	3.87E-06
SKCM	NEDD4L	3.56E-16	1.83E-05
THYM	NEDD4L	0.000126261	7.43E-08
CESC	NEDD4L	0.000144075	6.53E-07
LIHC	NEDD4L	8.66E-05	0.000421556
STAD	NEDD4L	9.39E-07	0.000548171
TGCT	NEDD4L	0.000743175	1.27E-11
PAAD	NEDD4L	8.09E-07	0.000744245
OV	NEDD4L	0.001010068	0.001837867
LGG	NEDD4L	0.01235798	1.18E-10
KIRP	NEDD4L	0.008044237	0.005736847
LAML	NEDD4L	0.004831584	0.015044435
HNSC	NEDD4L	0.000915919	0.022861088
ESCA	NEDD4L	0.070551927	0.00109635
PCPG	NEDD4L	0.018302933	0.082383888
COAD	NEDD4L	0.031388438	0.076398502
KICH	NEDD4L	0.078863218	0.039223729
MESO	NEDD4L	0.128425162	0.021833888
READ	NEDD4L	0.010343204	0.149422546
CHOL	NEDD4L	0.036388982	0.124041244
UCS	NEDD4L	0.182588143	0.171437062
DLBC	NEDD4L	0.405426195	0.021214432
ACC	NEDD4L	0.170649833	0.84407681

GBM	NEDD4L	0.912028877	0.251124592
THCA	NEDD4L	0.322032125	0.924467216
UVM	NEDD4L	0.36603136	0.891325801
